# Characteristics of Waveform Shape in Parkinson’s Disease Detected with Scalp Electroencephalography

**DOI:** 10.1523/ENEURO.0151-19.2019

**Published:** 2019-06-04

**Authors:** Nicko Jackson, Scott R. Cole, Bradley Voytek, Nicole C. Swann

**Affiliations:** 1 Department of Human Physiology, University of Oregon, Eugene, OR 97403; 2 Institute of Neuroscience, University of Oregon, Eugene, OR 97403; 3 Neurosciences Graduate Program, University of California San Diego, La Jolla, CA 92093; 4 Department of Cognitive Science, University of California San Diego, La Jolla, CA 92093; 5 Halıcıoğlu Data Science Institute, University of California San Diego, La Jolla, CA 92093; 6 Kavli Institute for Brain and Mind, University of California San Diego, La Jolla, CA 92093

**Keywords:** β, EEG, electrophysiology, motor cortex, Parkinson’s disease, waveform shape

## Abstract

Neural activity in the β frequency range (13–30 Hz) is excessively synchronized in Parkinson’s disease (PD). Previous work using invasive intracranial recordings and non-invasive scalp electroencephalography (EEG) has shown that correlations between β phase and broad-band γ (>50 Hz) amplitude [i.e., phase amplitude coupling (PAC)] are elevated in PD, perhaps a reflection of this synchrony. Recently, it has also been shown, in invasive human recordings, that non-sinusoidal features of β oscillation shape also characterize PD. Here, we show that these features of β waveform shape also distinguish PD patients on and off medication using non-invasive recordings in a dataset of 15 PD patients with resting scalp EEG. Specifically, β oscillations over sensorimotor electrodes in PD patients off medication had greater sharpness asymmetry and steepness asymmetry than on medication (sign rank, *p* < 0.02, corrected). We also showed that β oscillations over sensorimotor cortex most often had a canonical shape, and that using this prototypical shape as an inclusion criteria increased the effect size of our findings. Together, our findings suggest that novel ways of measuring β synchrony that incorporate waveform shape could improve detection of PD pathophysiology in non-invasive recordings. Moreover, they motivate the consideration of waveform shape in future EEG studies.

## Significance Statement

Diagnosis and long-term monitoring of Parkinson’s disease (PD) is mainly assessed via clinical rating scales which are subjective and can be imprecise. An objective measure of PD would be extremely valuable, especially one that could be acquired non-invasively. Here, we show, using scalp electroencephalography (EEG), that the non-sinusoidal shape of β (13–30 Hz) oscillations over sensorimotor cortex distinguishes PD patients on and off medication and patients off medication from controls. This change in waveform shape may reflect hypersynchronous input, possibly originating from basal ganglia. Thus, waveform shape is a putative non-invasive electrophysiological biomarker of PD state with potential utility for assessing treatments, monitoring disease, or diagnosis. This signature can be detected with a safe, affordable, and available method (i.e., EEG).

## Introduction

Parkinson’s disease (PD) is characterized by excessively synchronized neural activity in the β frequency range (13–30 Hz; Brown and Williams, 2005; Brown, 2007; Moran et al., 2008). Much of the work describing this phenomenon has come from invasive single unit and local field potential recordings from the human basal ganglia. Together, this body of work has formed a compelling narrative wherein motor system β synchrony, which can be detected using traditional β power in basal ganglia, is elevated in PD patients off medication and reduced by therapies like medication and deep brain stimulation (DBS; Kühn et al., 2006, 2008; Brown, 2007). This has motivated the idea that measures of brain physiology that reflect β synchrony could be potential “biomarkers” for PD pathophysiology. Such objective measures of PD symptoms would have enormous clinical potential for diagnosis, monitoring, and adjusting patient therapies. However, basal ganglia recordings can be difficult to acquire in humans and necessitate invasive approaches. Therefore, a cortical signature, which could be acquired non-invasively, or less invasively, could provide greater clinical utility.

While conventional signal processing measures, such as β power, have failed to reliably differentiate PD as a function of severity or diagnosis at the cortex (Stoffers et al., 2007; de Hemptinne et al., 2013, 2015; George et al., 2013; Swann et al., 2015; Malekmohammadi et al., 2018), novel metrics such as phase amplitude coupling (PAC) between β and broadband γ (50–150 Hz) have proven more promising. Specifically, PAC over motor cortex detected using electrocorticography (ECoG) is elevated in PD compared to other groups (de Hemptinne et al., 2013) and is reduced with DBS in a clinically-relevant manner (de Hemptinne et al., 2015; Malekmohammadi et al., 2018). Interestingly, following characterization of PAC with ECoG, it was demonstrated that elevated PAC can also be detected non-invasively with scalp electroencephalography (EEG; Swann et al., 2015). Moreover, PAC recorded with scalp EEG could differentiate PD patients on and off medication and differentiate PD patients off medication from healthy controls, at the group level.

Recently, increased attention has been given to novel waveform metrics, that may provide additional insights into neurophysiological mechanisms (Cole and Voytek, 2017). Using ECoG recordings, recent work has shown that β waveform shape, similar to PAC, differentiates PD patients on and off DBS (Cole et al., 2017). Specifically, PD patients off DBS had more sharp and asymmetric β activity, which was reduced on DBS. These waveform metrics may provide novel insights into underlying PD pathophysiology. Particularly, cortical β waveform shape may indicate the aggregate of synchronous inputs (perhaps from the basal ganglia via the thalamus) onto cortical pyramidal neurons (Sherman et al., 2016).

Given the potential utility of considering waveform shape, and the past literature that demonstrated that signatures reflecting PD pathophysiology initially characterized with ECoG are also detectable with EEG, we sought to investigate if waveform shape detected with scalp EEG might also be an electrophysiological biomarker for PD pathophysiology. Specifically, we tested if the shape of sensorimotor β activity in scalp EEG differed between PD patients on and off medication and PD patients compared to healthy age-matched control participants. We were especially interested in investigating how β waveform shape compares to PAC in its ability to distinguish medication state and what the strengths and weaknesses might be for each measure as a neurophysiological biomarker.

## Materials and Methods

### Participants

We analyzed a previously published data set, from another laboratory (George et al., 2013). This same data were analyzed in another published report which showed elevated PAC between β phase and broadband γ amplitude in PD patients off medication compared to the same patients on medication and compared to a group of healthy, age-matched, control participants (Swann et al., 2015). This dataset includes EEG data from 15 PD patients (eight female, mean age = 63.2 ± 8.2 years) on and off dopaminergic medication and 16 healthy, age-matched, control participants (nine female, mean age = 63.5 ± 9.6 years). All PD patients had been diagnosed by a movement disorder specialist at Scripps clinic in La Jolla, California. Participants were right-handed and provided written consent in accordance to the Institutional Review Board of the University of California, San Diego and the Declaration of Helsinki. For additional patient information, see George et al. (2013).

### Data collection

Data from patients on and off medication were collected on different days with a counterbalanced order. For the on-medication recordings, patients continued their typical medication regimen. For the off-medication state, patients discontinued medication use at least 12 h before the session. Control participants were tested once. EEG data were acquired using a 32-channel BioSemi ActiveTwo system, sampled at 512 Hz. Resting data were recorded for at least 3 min. During data collection, the participants were seated comfortably and told to fixate on a cross presented on a screen. Additional electrodes were placed lateral to and below the left eye to monitor eye blinks and movements. Participants also completed several other assessments described in the previously published report (George et al., 2013), which were not analyzed here.

### Data preprocessing

Data were preprocessed in MATLAB using custom scripts and EEGLAB functions (Delorme and Makeig, 2004). In brief, first, each electrode’s mean was removed and then a common average reference was applied. Excessively noisy electrodes were excluded. The data were then high pass filtered at 0.5 Hz to remove low frequency drift (using a two-way FIR filter, eegfilt; Delorme and Makeig, 2004). Then, data were manually examined for artifacts (eye blinks and movements, muscle activity, electrical noise, and other sources of noise), and indices containing these events were flagged for rejection. to avoid filtering over non-continuous data, rejection regions were excluded after filtering (as described below). A previously published report examining the same data (Swann et al., 2015) used a current source density (CSD) referencing montage to examine PAC (Kayser and Tenke, 2006a,b; Kayser, 2009). We opted not to use this approach here since this montage might produce more unpredictable phase inversions and complicate interpretation of waveform shape. Thus, to be consistent, we used an average reference approach for all measures. However, for direct comparison with the previous publication (Swann et al., 2015) we implemented a CSD referencing approach as a confirmatory analysis (CSDtoolbox in MATLAB, spline flexibility = 3; Kayser and Tenke, 2006a,b; Kayser, 2009).

We focused predominantly on electrodes closest to sensorimotor cortex (C3 and C4). To compare data from these electrodes to recordings more likely to be impacted by residual electromyogram (EMG) artifact we also examined the electrodes closest to the temporalis muscles (F7 and F8; Swann et al., 2015). Unless otherwise specified, statistical testing focused on a “composite” sensorimotor signal where measures from C3 and C4 were calculated separately and then results were averaged for each participant, such that each participant contributed one data point, before performing statistical testing across participants. The same procedure was used for electrodes closest to the temporalis muscles.

### Data analysis

The following measures were calculated using Python code that were modified from (Cole et al., 2017), unless otherwise specified. Details are described below for each measure. Based on the previous publications, we focused our analysis on the β frequency range (de Hemptinne et al., 2015; Swann et al., 2015; Cole et al., 2017). However, we did compute average power spectral density (using the pwelch function in MATLAB, window = 256, overlap =128, nFFT = 450) to verify that an oscillation was indeed present in the β frequency range and to determine if prominent oscillations in other frequency ranges were also present.

### Code accessibility

The original code can be found at https://github.com/voytekresearch/Cole_2017. The code we used for this manuscript can be found at https://github.com/SwannLab/Jackson_2019 and is also available as Extended Data Code 1.

10.1523/ENEURO.0151-19.2019.ed1 Extended Data 1 Code used for this manuscript. Also available at https://github.com/SwannLab/Jackson_2019. Download Extended Data 1, ZIP file.

### Sharpness ratio

**Figure 1.**
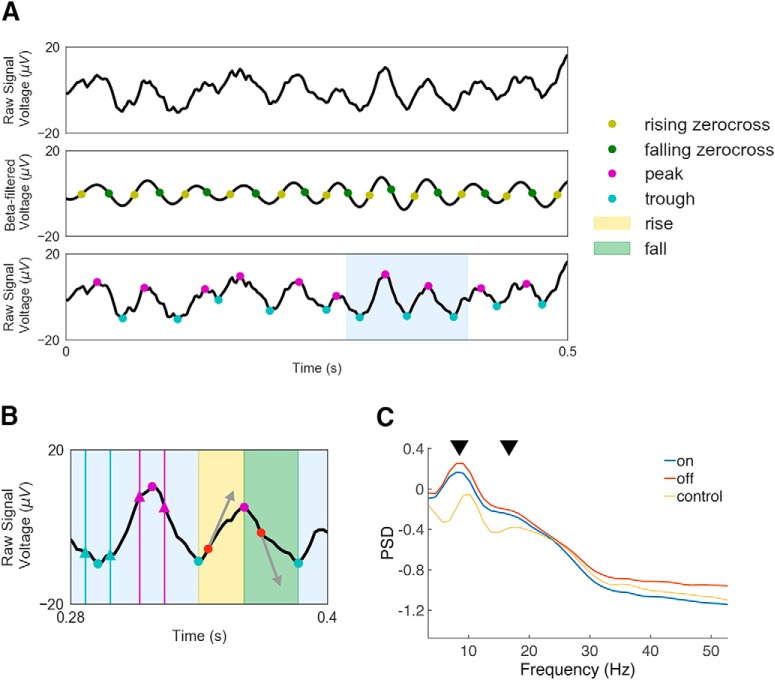
Schematic summarizing waveform shape calculations. ***A***, First rising and falling zerocrossings were identified from the β-filtered signal. Then, peaks and troughs that lie between adjacent zerocrossings were found in the raw signal. ***B***, Peak sharpness was calculated as the mean of the difference between the peak (purple circle) and the voltage points three samples before and after the peak (purple triangles). The trough sharpness was calculated in a similar fashion, indicated with blue circles and triangles similar to the peaks analysis. The yellow area, between a trough and subsequent peak, indicates an area where rise steepness is determined, while the green area, between a peak and subsequent trough, indicates an area where decay steepness was found. The maximum slope (shown with arrows) for each of these shaded regions (red circles) was derived as rise and decay steepness and then the ratio was taken to find steepness ratio, as described in Materials and Methods. Figure modeled after Cole et al. (2017), but here is shown with the present EEG data. ***C***, Average power spectral density plots for each group. Plots confirm that there is a β oscillation as well as a prominent α/μ oscillation (each indicated with black arrow).

We calculated sharpness ratio using previously defined methods (Cole et al., 2017). In brief, each signal was filtered between 13 and 30 Hz using a window-method based FIR filter (scipy.filter.firwin, order = 231 ms). Indices of rising and falling zerocrossings (i.e., voltage sign-changes) were identified in this filtered signal. Next, returning to the raw signal, the indices of the maximum voltage (the “peak”), and minimum voltage (the “trough”), between zerocrossings were found. Once peaks and troughs were identified, those that fell in regions previously marked for rejection due to artifacts were excluded from further analysis. Peak sharpness was defined as the mean of the voltage difference between the peak and three data points before and after the peak. Trough sharpness was calculated in an analogous way. Three sample points corresponds to ∼5.9 ms of data. Similar to a previous report (Cole et al., 2017), calculating sharpness ratio using different widths produced similar results. Sharpness ratio was defined as the log of the maximum ratio of either mean peak sharpness to mean trough sharpness, or mean trough sharpness to mean peak sharpness (Cole et al., 2017). The maximum was taken so that logged ratios were positive. See Figure 1 for schematic explaining experimental methods.

### Steepness ratio

Steepness ratio was also calculated according to previously published methods (Cole et al., 2017). Rise steepness, between a trough and a subsequent peak, was defined as the maximum value of the first derivative, or the greatest slope, of that portion, of the signal. Similarly, decay steepness was defined as the maximum of the absolute value of the slope between each peak and subsequent trough. Steepness ratio was calculated as either the ratio of mean decay steepness to mean rise steepness or vice versa, such that the logged ratio was positive.

### PAC

PAC, which reflects correlations between β phase and broad-band γ amplitude, was calculated using the normalized modulation index metric (Özkurt and Schnitzler, 2011), with the open-source package, pacpy, which can be found at https://github.com/voytekresearch/pacpy. In brief, using a FIR filter with a hamming window, β and γ components were extracted from the signal. Beta was filtered between 13 and 30 Hz with a filter order of 231 ms using the function “firwin” in python. Gamma was filtered using the same filter between 50 and 150 Hz with a filter order of 240 ms. After filtering, regions that had been marked as containing artifacts were removed. Beta phase and γ amplitude time series were calculated by extracting the angle and amplitude of the Hilbert transform, respectively. Note that PAC results have already been published using these data (Swann et al., 2015). However, we repeat this analysis here for direct comparison with the waveform shape metrics. Note also that here we used an alternative method to calculate PAC to be consistent with previous comparisons between waveform shape and PAC (Cole et al., 2017). However, results were similar between methods, except where noted.

### Peak-to-trough ratio and rise-to-fall ratio

Sharpness ratio and steepness ratio quantify overall asymmetry but do not characterize the specific peak/trough or rise/fall contributions to this asymmetry. To address this, and further characterize waveform shape, we calculated peak-to-trough ratio (analogous to sharpness) and rise-to-fall ratio (analogous to steepness). Peak-to-trough ratio is the log of mean peak sharpness divided by mean trough sharpness, so that a positive peak-to-trough ratio indicates relatively sharper peaks while a negative ratio indicates relatively sharper troughs. Similarly, rise-to-fall ratio is the log of the mean rise steepness divided by the mean fall steepness, and a positive rise-to-fall ratio signifies a relatively steeper rise and a negative rise-to-fall ratio indicates a relatively steeper decay. Because these measures differentiate peaks/troughs and rise/fall, they allow typification of waveform shape into four distinct categories (i.e., negative vs positive peak-to-trough ratio and rise-to-fall ratios.

### Statistical tests

For across group comparisons we used a non-parametric Wilcoxon signed rank test for all comparisons of patients on and off medication and Wilcoxon rank sum test to compare patients off medication to controls. Spearman correlation coefficients were calculated to compare similarities between PAC and waveform shape-based metrics. A false discovery rate (FDR) correction was applied for multiple comparisons (Benjamini and Hochberg, 1995).

We also calculated effect size for each waveform metric. Effect size (Cohen’s *d*) quantitatively measures the magnitude of an effect (Cohen, 1998). For these calculations, PAC was log-scaled to ensure normalization. Sharpness ratio and steepness ratio were already log scaled as part of their standard analysis.

## Results

### Waveform metrics differentiate PD medication state

**Figure 2.**
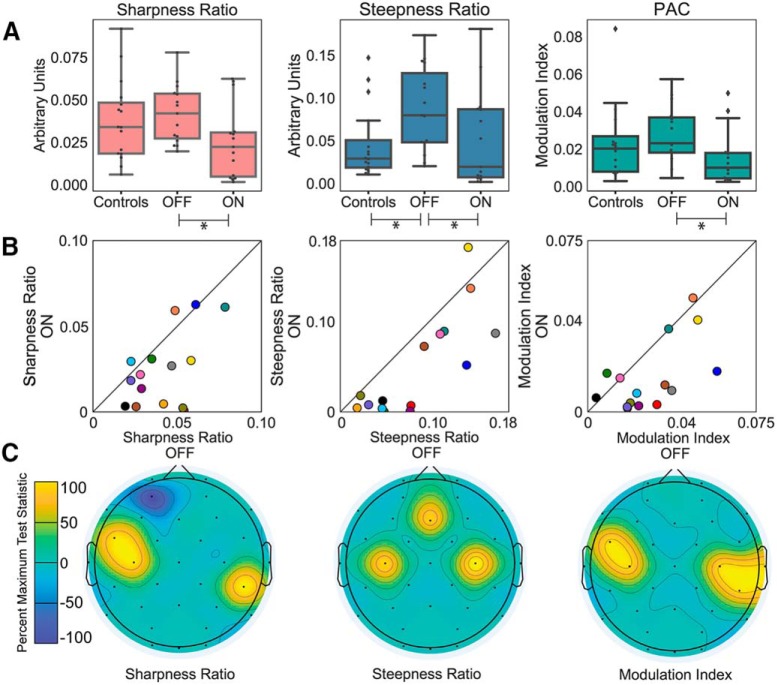
Sharpness ratio, steepness ratio, and PAC all decreased with medication in PD patients. ***A***, Box plots of each measure for patients off medication, on medication, and control participants. Asterisk represents FDR corrected significance at *p* < 0.05. Note that the on versus off medication comparison was a paired comparison (Wilcoxon sign rank) whereas the off medication versus control comparison was unpaired (Wilcoxon rank sum). ***B***, Individual data for each measure and each patient on and off medications. Each color corresponds to a different participant. Diagonal lines represent unity. ***C***, Scalp topography for on versus off medication for each measure. For each electrode, test statistics below the critical value, indicating significance, were set to zero. Test statistics above the critical value were rescaled and normalized to the percentage of the most significant test statistic, such that 100 would reflect the most significant test statistic.

Medication reduced beta waveform sharpness ratio (*p* = 0.012, FDR corrected; Fig. 2) and steepness ratio (*p* = 0.012, FDR corrected; Fig. 2) in PD patients over sensorimotor electrodes. Notably, sharpness ratio and steepness ratio are higher for waves with asymmetrical waveforms (e.g., sawtooth or arched waves), suggesting that medication flattened waveforms or made them more symmetric. PAC also decreased with medication (*p* = 0.017, FDR corrected; Fig. 2), as has been previously published. Note that these changes were in the context of significant clinical improvement with medication as measured by Unified Parkinson’s Disease Rating Scale measures (Wilcoxon signed rank test statistic = 3.5, *p* = 0.003). Because many clinical EEGs use a bipolar montage, we also calculated these measures using a bipolar scheme (C3-CP1 and C4-CP2) and found very similar results (*p* < 0.004, FDR corrected for each of the three measures examined). This demonstrates that these measures could be derived from even just two recording electrodes (if recording unilaterally). However, to minimize phase inversions, we used the average reference montage for our additional analyses here.

Data from electrodes closest to the temporalis muscles (F7 and F8) were also examined to compare the sensorimotor results to electrodes more likely to be contaminated by muscle activity and address our concerns that differences in muscle activity may have been contributing to differences. However, none of these measures differed between patients on and off medication over these electrodes: sharpness ratio, *p* = 0.73, uncorrected, steepness ratio, *p* = 0.394, uncorrected PAC, *p* = 0.191, uncorrected (as was previously published, using an alternative PAC calculation and alternative referencing scheme). Results were similar for T7 and T8, also near temporalis muscles (*p* > 0.05, uncorrected for all comparisons). Additionally, we calculated scalp topographies (using topoplot.m in EEGLAB) across all electrodes to show which electrodes differentiated the off versus on medication state, for visualization purposes, and found that topographies were consistent with a sensorimotor origin (Fig. 2*C*).

We also examined PD patients off medication compared to control participants. There was no significant difference between these groups for beta waveform sharpness ratio (*p* = 0.363, uncorrected), however steepness ratio did distinguish patients from controls (*p* = 0.033 using the average referencing scheme and *p* = 0.028 for a bipolar referencing scheme, both FDR corrected). Somewhat surprisingly there was also no significant difference between PD patients off medication and control participants for the PAC calculation (*p* = 0.236, uncorrected). However, a previous publication testing these same data, but using a CSD referencing scheme (which is designed to increase spatial specificity), and an alternative method for calculating PAC (Tort et al., 2008) did find a significant difference between PD patients off medication and control participants (Swann et al., 2015). When we tested the CSD referencing scheme using the current method for PAC calculation, there was a significant difference between patients off medication and controls (*p* = 0.022), confirming findings from the previous publication which suggest PAC differences between patients and controls were sensitive to referencing scheme and were most apparent for approaches which isolate spatial sources (Swann et al., 2015). Note that we chose not to use the CSD reference for the waveform shape calculations since this scheme might cause unpredictable phase inversions. Thus, this CSD approach was considered only as a confirmatory analysis to test whether alternative methods to calculate PAC or referencing schemes impacted the comparison of patients off medication compared to controls.

While we focused on the β range based on the previous publications, we also plotted average power spectral density plots for each group to verify that an oscillation in β was indeed present (Fig. 1*C*). This is important because analyzing the raw signal as is done for the sharpness and steepness measures is not precise if there is no dominant oscillation present. These plots demonstrated that there was indeed an oscillation present in β, as would be expected in sensorimotor EEG. However, unsurprisingly there was also a μ/α oscillation in the 8–12 Hz range (Pfurtscheller, 1981). Thus, we repeated our analyses for the μ/α range and have reported those results in Tables 1, 2. We found these values to be overall similar to those we found for β, with the exception of PAC, which was non-significant for μ/α, while being significant for β. Indeed, for PAC, the difference between off medication and on medication in β was significantly larger than the difference in μ/α for the same comparison (Wilcoxon signed rank test statistic = 11, *p* = 0.005). This is perhaps not surprising since PAC is the only measure which was not derived from the raw signal (but rather filtered signals). Given that β was the focus of the previous publications (de Hemptinne et al., 2015; Swann et al., 2015; Cole et al., 2017), and here the β results were more robust for PAC and the effect size was greater for β for nearly all measures examined (Tables 1, 2), we focused on β for the remainder of the analyses. However, we do report μ/α results in Tables 1, 2 and also correct all the *p* values in each table for all measures (sharpness ratio, steepness ratio, and PAC) and all frequencies with a clear oscillation in the power spectral density plots (μ/α and β) using an FDR correction as reported in the table.

**Table 1.** Comparisons of patients off medication versus on medications

Frequency range	Metric	Test statistic (signed rank)	*p* value (FDR corrected)	Effect size (d)
μ/α (8–12 Hz)	Sharpness ratio	20	0.023 (0.0278)	0.70
	Steepness ratio	12	0.006 (0.0121)	0.60
	PAC	30	0.088 (0.0881)	0.51
β (13–30 Hz)	Sharpness ratio	12	0.006 (0.0121)	0.86
	Steepness ratio	9	0.004 (0.0121)	0.68
	PAC	15	0.011 (0.0166)	0.83

Values are shown for both μ/α and β based on a priori hypotheses (β) and the average power spectral density plots (μ/α and β; Fig. 1*C*). Multiple comparisons correction (FDR) is applied including correction for all comparisons in the table.

**Table 2.** Comparisons patients off medication versus control participants

Frequency range	Metric	Test statistic (rank sum)	*p* value (FDR corrected)	Effect size (d)
μ/α (8–12 Hz)	Sharpness ratio	1.03	0.304 (1)	0.28
	Steepness ratio	2.53	0.011 (0.0331)	0.87
	PAC	2.17	0.030 (0.0602)	0.76
β (13–30 Hz)	Sharpness ratio	0.91	0.3631 (1)	0.19
	Steepness ratio	2.53	0.011 (0.0331)	0.88
	PAC	1.19	0.236 (1)	0.27

Values are shown for both μ/α and β, as in Table 1. Multiple comparisons correction (FDR) is applied including all comparisons in the table.

Overall, the difference between patients on and off medication was robust over sensorimotor cortex with significant differences for each of our measures in the beta range. In contrast, measures over electrodes more likely to be contaminated by EMG artifact (i.e., closest to temporalis muscles) did not differentiate groups for any measure. Therefore, we believe that our findings are less likely to be driven by muscle artifact.

The comparison between patients off medication and controls was less clear with only beta steepness ratio distinguishing the groups (and PAC calculated using a CSD reference as was previously reported; Swann et al., 2015).

### Relationships between sharpness/steepness ratio and PAC

**Figure 3.**
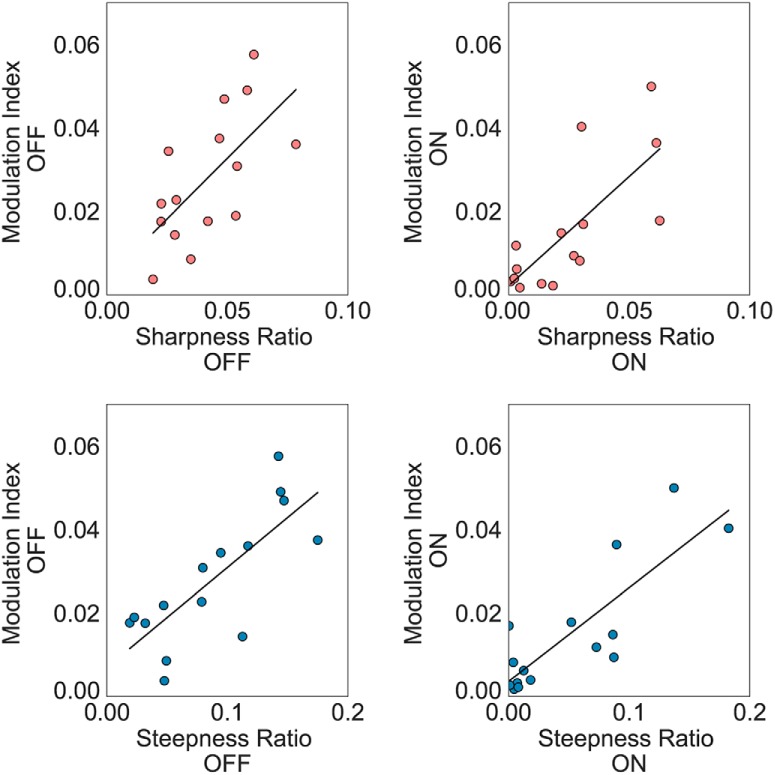
Both sharpness ratio and steepness ratio over sensorimotor cortex correlate with PAC in both medication states.

To address how waveform shape and PAC might relate to one another and probe whether they may be reflecting the same underlying process or be picking up on different aspects of pathophysiology, we examined relationships between the waveform shape measures and PAC. Like the previous report using invasive recordings (Cole et al., 2017), sharpness ratio and PAC were correlated in both on and off medication states in sensorimotor electrodes (off medication: *r* = 0.70, *p* = 0.004; on medication: *r* = 0.74, *p* = 0.002; Fig. 3). Likewise, steepness ratio and PAC are also highly correlated for both medication states (off medication: *r* = 0.74, *p* = 0.002; on medication: *r* = 0.68, *p* = 0.006).

### Waveform shape

**Figure 4.**
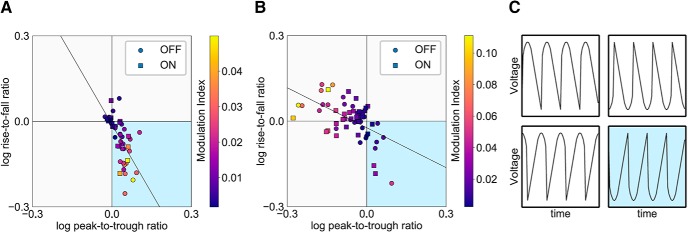
Sensorimotor cortex recordings have a canonical shape. ***A***, Peak-to-trough ratio versus rise-to-fall ratio over sensorimotor electrodes (C3 and C4) shown with individual participants/electrodes. Circles represent off medication, while squares represent on medication. The color map corresponds to PAC values. Only patient data are shown, but control data followed a similar pattern. ***B***, Peak-to-trough versus rise-to-fall ratio in electrodes closest to temporalis muscles (F7 and F8) shown separately. Note the scale of the PAC color map is different from in ***A***. ***C***, Representative waveform shape for each quadrant. The sensorimotor data falls mainly in Q4 (blue). This corresponds to sharper peaks and steeper decays.

Steepness and sharpness measures can be considered together to categorize waveforms into different shapes. Previous reports have shown that certain shapes may dominate for certain recording types (Cole et al., 2017). Here, we observed qualitative differences in waveform shape in sensorimotor electrodes which differed from electrodes closer to temporalis muscles (F7 and F8; Fig. 4). In general, sensorimotor electrode oscillations had a sharper peak with a steeper decay, falling in quadrant 4 (Q4) in Figure 4. In contrast, electrodes closest to temporalis muscles had a more variable waveform shape pattern, perhaps consistent with contributions from both EEG and EMG, and/or the general lack of a consistent dominant waveform pattern. Note that here, our goal was to show qualitatively the overall shape of the data, so we have included in Figure 4 data from each electrode (C3 and C4) as well as data from patients on and off; thus, dependent samples are included. Although not shown, control participant data exhibited the same pattern (i.e., sensorimotor electrode data fell mainly in Q4).

We next tested whether including only sensorimotor data that fell in Q4 would strengthen medication state differentiation (since this quadrant captured the putative dominant shape of the sensorimotor activity). The rationale is that if this shape is a canonical feature of sensorimotor EEG data, using it as an inclusion criteria might exclude data without a canonical sensorimotor “shape” (i.e., perhaps because β power is lower or residual EMG may be obscuring activity). For this analysis, patients were only included if they had data on and off medication that fell in Q4. Out of 15 patients, nine patients met this criterion for C3 and eight met the criterion for C4. When data from both C3 and C4 fell within Q4 (eight patients), measures were calculated for each electrode separately and then averaged so that each patient contributed 1 data point for the effect size calculation, analogous to the above analyses. When only one electrode was available (one patient), measures from just this electrode were used for the effect size analysis. Using this approach there were nine total Q4 observations for each waveform metric. We compared effect size when all data were used for the calculation, to the effect size when only data from Q4 were included. Effect size increased for all measures, at least marginally, when using the Q4 shape as an inclusion criterion (Table 3).

**Table 3.** Effect size (Cohen’s *d*) for all patients and for Q4 patients, on versus off medication

Metric	All	Q4
Sharpness ratio	0.86	0.95
Steepness ratio	0.68	0.70
PAC	0.75	0.83

All metrics were log scaled.

## Discussion

Converging work has shown that PD is associated with excessive β synchrony. Here, we show that this pathophysiological synchrony is manifest in a change in waveform shape which can be detected at the scalp. Specifically, we show waveforms are more asymmetric for PD patients off medication and that this is reduced with medication. Further, we show that including only data with a canonical arched waveform shape, consistent with a sensorimotor “μ” shape, increased effect size, at least marginally, for all metrics examined. This suggests the consideration of waveform shape could improve the efficacy of non-invasive PD biomarkers. Importantly, this difference in waveform shape occurred in the context of a lack of a consistent difference in conventional spectral power in the β range over sensorimotor cortex in previous publications examining PD (Stoffers et al., 2007; de Hemptinne et al., 2013, 2015; Malekmohammadi et al., 2018), including two which analyzed the same dataset we examined here (George et al., 2013; Swann et al., 2015). This underscores the general importance of including novel metrics, such as waveform shape, in analyses of electrophysiological data, including scalp EEG.

### Mechanisms of waveform shape asymmetry and PAC

Converging evidence suggests that PAC is elevated in untreated PD patients compared to treated (de Hemptinne et al., 2015; Swann et al., 2015; Malekmohammadi et al., 2018). It also emerges during the induction of severe parkinsonian symptoms in non-human primate models, and is related to disease severity (Devergnas et al., 2017). However, the etiology of PAC is unclear. One potential mechanism of PAC is that it describes a relationship between lower frequency activity and high frequency broadband amplitude, a putative surrogate for spiking (Manning et al., 2009). Thus, synchronous cortical input in β (perhaps from basal ganglia via thalamus) may bias the probability of neural spiking. This is supported in PD, by the observations of unit spiking coupled to β local field potentials in basal ganglia recordings (Moran et al., 2008). Additionally, it was recently demonstrated in human subthalamic nucleus that high PAC is indeed associated with locking of unit firing to β oscillations (Meidahl et al., 2019). However, several other phenomena can also lead to increased PAC, including periodic sharpness or transients (Kramer et al., 2008; Aru et al., 2015). Comparably, sensorimotor waveform shape may reflect synchronous inputs (Sherman et al., 2016) perhaps from basal ganglia (Nini et al., 1995; Sharott et al., 2005; Brown, 2007; Mallet et al., 2008), that are not necessarily associated with spiking. Excessive basal ganglia-thalamocortical loop synchrony may constrain neurons in an inflexible pattern, prevent changes necessary for dynamic behavior, and impair neural communication by exhausting “neural bandwidth” (Reyes, 2003; de Hemptinne et al., 2013; Swann et al., 2015; Voytek and Knight, 2015; Cole et al., 2017). We suspect that waveform shape metrics and PAC (and likely other measures elevated in untreated PD such as global β coherence across electrodes; Silberstein et al., 2005) are imperfect ways of measuring the same underlying pathophysiology, excessive β synchrony and neural entrainment. This synchrony exists in the healthy state, but is amplified throughout motor networks, both within and between individual structures in PD. Dopaminergic medication reduces this excessive synchrony, in conjunction with improvement of the Parkinsonian state, which is reflected in the reduction of PAC, sharpness ratio, and steepness ratio (de Hemptinne et al., 2015).

### Differences and similarities between measures

Consistent with previous findings in ECOG recordings (Cole et al., 2017), waveform shape (sharpness ratio and steepness ratio) is correlated with PAC, indicating that they may be picking up similar characteristics. Indeed, Cole and colleagues showed remarkable correspondence between broadband amplitude measured in PAC and non-sinusoidal aspects of waveform shape. Therefore, it could be that elevated PAC may be better described as a change in waveform shape, a more parsimonious explanation of the signal (i.e., a measure of one periodic process, β, rather than two, β and γ; Cole et al., 2017). Alternatively (or additionally) PAC and waveform shape may be correlated because they both relate to PD severity or PD pathophysiology.

Despite high correlations between measures, PAC could not be completely explained by waveform shape in our data, and the measures show differences in detection ability, especially for PD patients off medications compared to healthy controls. This suggests that different measures may have different strengths and weaknesses, even if detecting the same underlying pathophysiology.

### Possible ways to improve the electrophysiology biomarker

The measures examined here are only robust when there is a dominant waveform present. While this can be accessed by examining power spectral density (as we performed here), examining presence of spectral power for specific frequencies with cycle-by-cycle specificity would offer improved precision. Such algorithms are currently in development or becoming newly available (Cole and Voytek, 2018).

Considering waveform shape as an inclusion-criteria for our group analyses improved the effect size, suggesting that development of a method to extract waveforms of a specific shape from a signal might result in an even more powerful biomarker. This may be especially important for EEG data, which is more likely to suffer from lower signal-to-noise ratio. Therefore, developing a method to select only data with specific shapes, perhaps by building a filter which only extracts signals with a canonical sensorimotor shape (i.e., a sharp peak and steep fall), might improve classification since it would isolate data with prototypical patterns consistent with a sensorimotor origin. Additionally, this filter could also be used to detect oscillatory bursts with this shape within the β frequency range (Tinkhauser et al., 2017) or to index the cycle-to-cycle variation in a β signal (Torrecillos et al., 2018). Alternatively, principal component analysis could be used to create an optimal waveform shape metric that is comprised of weighted components of each waveform measure.

Nevertheless, it is promising that measures of β waveform shape could distinguish PD patients off versus on medication and in some cases, patients compared to controls, even with short duration recordings and relatively straightforward analytic methods.

### Uses for a non-invasive electrophysiological biomarker

Non-invasive EEG enables easy and inexpensive measurements in patients and allows for comparisons to healthy controls. Since waveform metrics robustly index medication status using EEG, they can potentially be used as an objective metric reflecting PD state. Waveform shape metrics may be particularly conducive to real-time measures, since they can theoretically be derived from shorter periods of data compared to PAC, for instance. There are many applications for an objective, non-invasive measure of PD state. For instance, such a measure could be used by clinicians to calibrate medications or by patients to determine when to take a medication dose. An objective measure could also be useful for DBS updates, with possible utility for adjusting DBS in real-time (Little et al., 2013; Swann et al., 2018), or for helping find optimal DBS settings, by providing an objective measure which could be used for “automatic programming.” This could optimize the time-consuming and sometimes imprecise process of programming in clinic which usually relies on trial and error assessment. These approaches may be especially useful for PD patients who lack easy access to neurologists, especially those who are mobility impaired or live in remote areas. With increases of wearable technology, one could imagine that acquiring these measures could become increasingly easy even in patients’ own homes.

### Limitations

For the on-medication recordings, patients had variable amounts of time since their last dose, which could have contributed to some of the variability in our results. However, the fact that differences were still apparent despite this variability, suggests that the findings were robust. Indeed, this variability and associated physiology may be closer to what patients would experience in daily life, reinforcing the translational potential of these approaches. Furthermore, having a small sample size with heterogeneous patients may have added to sample variability. Validation in a larger sample size of patients who have been carefully clinically characterized will be necessary, particularly to evaluate possible correlations to particular clinical symptoms, disease severity, and other clinical criteria on an individual patient level.

Although EEG has an obvious advantage in terms of safety and accessibility compared to invasive recordings, it also has a lower signal-to-noise ratio, poor spatial resolution, and can be impacted by ocular and muscle artifacts. Nevertheless, EEG has greater translational potential and here we demonstrate robust features, even with these methodological shortcomings.

### Conclusion

In PD, basal ganglia thalamocortical loops are excessively synchronized. We show that this synchrony can be detected cortically using scalp EEG as elevated PAC and altered waveform shape in PD patients off medication compared to on. Furthermore, we have shown that considering waveform shape, specifically using a particular characteristic shape as an inclusion criterion, increased effect size, suggesting that waveform shape might be useful for optimizing classification power for a non-invasive electrophysiological biomarkers of PD. Finally, our findings highlight the importance and value of considering waveform shape for future EEG studies.

## Synthesis

Reviewing Editor: Satu Palva, University of Helsinki Decisions are customarily a result of the Reviewing Editor and the peer reviewers coming together and discussing their recommendations until a consensus is reached. When revisions are invited, a fact-based synthesis statement explaining their decision and outlining what is needed to prepare a revision will be listed below. Note: If this manuscript was transferred from JNeurosci and a decision was made to accept the manuscript without peer review, a brief statement to this effect will instead be what is listed below.

The manuscript was accepted after editorial review.
